# 3D morphology-based clustering and simulation of human pyramidal cell dendritic spines

**DOI:** 10.1371/journal.pcbi.1006221

**Published:** 2018-06-13

**Authors:** Sergio Luengo-Sanchez, Isabel Fernaud-Espinosa, Concha Bielza, Ruth Benavides-Piccione, Pedro Larrañaga, Javier DeFelipe

**Affiliations:** 1 Computational Intelligence Group, Departamento de Inteligencia Artificial, Escuela Técnica Superior de Ingenieros Informáticos, Universidad Politécnica de Madrid, Campus Montegancedo, Madrid, Spain; 2 Laboratorio Cajal de Circuitos Corticales, Centro de Tecnología Biomédica, Universidad Politécnica de Madrid, Campus Montegancedo, Madrid, Spain; 3 Centro de Investigación Biomédica en Red Sobre Enfermedades Neurodegenerativas, Instituto de Salud Carlos III, Madrid, Spain; 4 Departamento de Neurobiología Funcional y de Sistemas, Instituto Cajal (CSIC), Madrid, Spain; Goethe University, GERMANY

## Abstract

The dendritic spines of pyramidal neurons are the targets of most excitatory synapses in the cerebral cortex. They have a wide variety of morphologies, and their morphology appears to be critical from the functional point of view. To further characterize dendritic spine geometry, we used in this paper over 7,000 individually 3D reconstructed dendritic spines from human cortical pyramidal neurons to group dendritic spines using model-based clustering. This approach uncovered six separate groups of human dendritic spines. To better understand the differences between these groups, the discriminative characteristics of each group were identified as a set of rules. Model-based clustering was also useful for simulating accurate 3D virtual representations of spines that matched the morphological definitions of each cluster. This mathematical approach could provide a useful tool for theoretical predictions on the functional features of human pyramidal neurons based on the morphology of dendritic spines.

## Author summary

Dendritic spines of pyramidal neurons are the targets of most excitatory synapses in the cerebral cortex and their morphology appears to be critical from the functional point of view. Thus, characterizing this morphology is necessary to link structural and functional spine data and thus interpret and make them more meaningful. We have used a large database of more than 7,000 individually 3D reconstructed dendritic spines from human cortical pyramidal neurons that is first transformed into a set of 54 quantitative features characterizing spine geometry mathematically. The resulting data set is grouped into spine clusters based on a probabilistic model with Gaussian finite mixtures. We uncover six groups of spines whose discriminative characteristics are identified with machine learning methods as a set of rules. The clustering model allows us to simulate accurate spines from human pyramidal neurons to suggest new hypotheses of the functional organization of these cells.

## Introduction

It is known that the dendritic spines (for simplicity’s sake, spines) of pyramidal neurons are the targets of most excitatory synapses in the cerebral cortex [1]. Numerous studies suggest that spine shape could determine their synaptic strength and learning rules and is also related to the storage and integration of excitatory synaptic inputs in pyramidal neurons [2]. Quantitative analyses have demonstrated strong correlations between spine morphological variables and synaptic structure. Specifically, the spine head volume in the neocortex is correlated with the area of the postsynaptic density (PSD) [3]. Both parameters are highly variable across spines. Interestingly, however, the spine head volume (like the total spine volume) is positively correlated with the PSD area, and there is a remarkably small variance. Moreover, PSD area is correlated with the number of presynaptic vesicles, the number of postsynaptic receptors and the readily-releasable pool of transmitters. By contrast, the length and diameter of the spine neck is proportional to the extent to which the spine is biochemically and electrically isolated from its parent dendrite [4–8]. Also, it has been shown that larger spines can generate larger synaptic currents than smaller spines [9]. Furthermore, dendritic spines are dynamic structures with volume fluctuations that appear to have important implications for cognition and memory [10–13]. Therefore, spine morphology appears to be critical from the functional point of view (for a review, see [14]).

There are a wide variety of spine morphologies, especially in the human cortex [15]. While many different classifications of spines have been proposed on the basis of their morphological characteristics, the most widely used was proposed by Peters and Kaiserman-Abramof [16] which groups spines into three basic categories—thin, mushroom and stubby spines—and an additional category—filopodia. However, it has also been argued that the large diversity of spine sizes reflects a continuum of morphologies rather than the existence of discrete groups [3]. Automatic clustering techniques over 2D spine representations have recently been used [17,18] to address this argument with the aim of avoiding the subjectivity and bias involved in manual analysis. Both studies consider that some spines cannot be clearly assigned to one of Peters and Kaiserman-Abramof’s classes because these spines are transitions between shapes.

However, the geometry of spines can be more accurately determined by means of 3D reconstructions, since many morphological features are not taken into account in 2D.

Ideally, 3D reconstruction using electron microscopy serial sections is the gold standard to obtain accurate estimations of the geometry of spines. However, a relatively low number of spines (at best in the order of a few hundred) can be reconstructed in 3D using electron microscopy in a reasonable time period, and these reconstructions can only be carried out in small segments of the dendritic arbor of the neurons. Furthermore, the quality of electron microscopy when using human brain tissue is usually suboptimal due to technical constraints. On the contrary, fluorescent labeling of neurons and the use of high power reconstruction with confocal microscopy (or other techniques) allow the visualization of thousands of spines with high quality along the dendritic arbor (apical and basal dendrites). Thus, in this study, we used a large, quantitative database of completely 3D-reconstructed spines (7,916) of human cortical pyramidal neurons—using intracellular injections of Lucifer Yellow in fixed tissue—to further characterize spine geometry [15].

Here we proposed a new set of 54 features. They were selected so as to unambiguously approximate the 3D shape of spines, enabling 3D simulation of spines. A probabilistic clustering grouped the 3D reconstructed human spines according to the selected set of morphology-based features. The best number of groups for probabilistic clustering based on the Bayesian information criterion was six groups of human spines. The interpretation of the clusters in terms of their most discriminative characteristics relied on the rules generated automatically by a rule induction algorithm. Since previous studies have shown that there are selective changes in dendritic and spine parameters with aging and dendritic compartments [15,19–21], we also explored the distributions of the groups according to dendritic compartment, age and distance from soma to further characterize possible variations according to these parameters. Finally, we present a stochastic method designed to simulate biologically feasible spines according to the probabilities defined by the clustering model. We introduce a procedure to shape simulated spines generating their 3D representations. To the best of our knowledge, this is the first attempt to fully characterize, model and simulate 3D spines.

## Results

### Clustering of spines into six different groups according to a selected set of geometrical features

**Fig 1:**
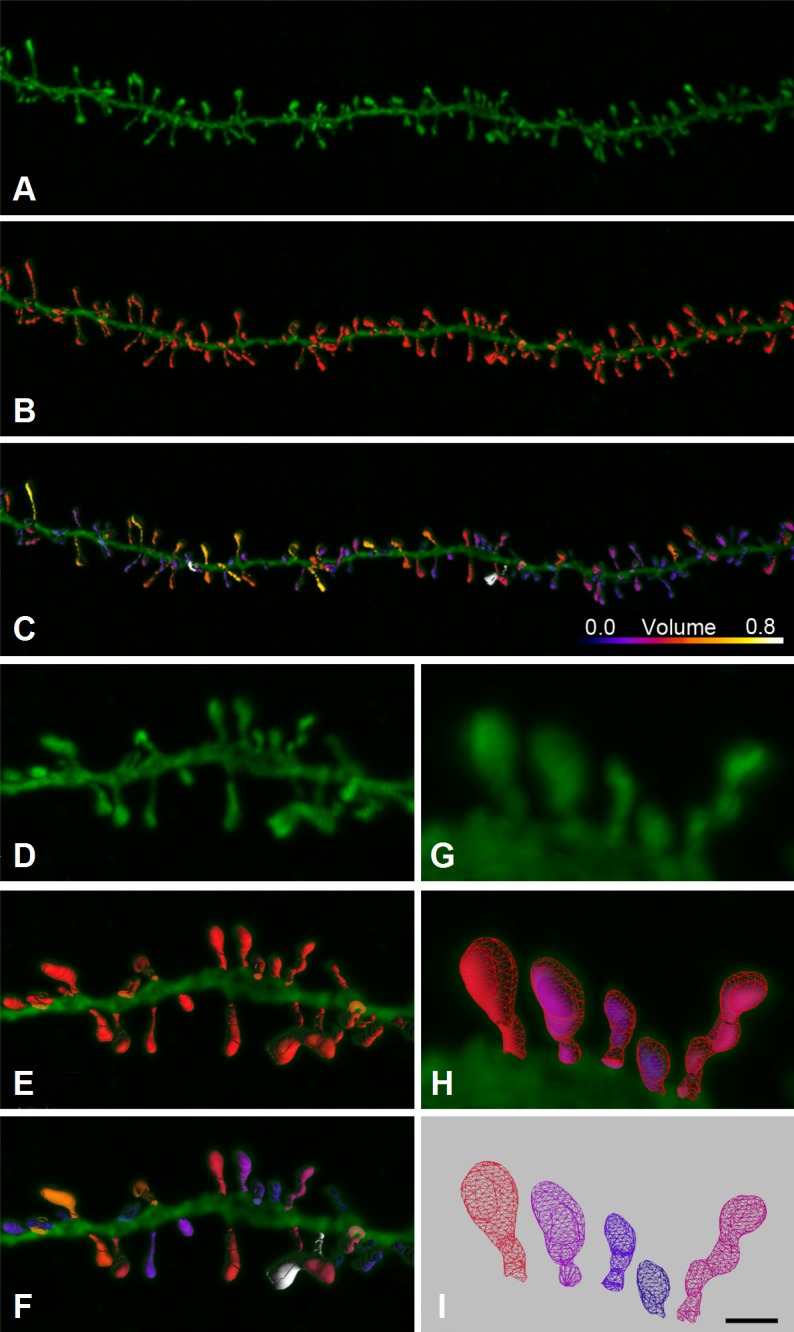
3D reconstructions of human dendritic spines. **(A)** Confocal microscopy z-projection image showing a horizontally projecting basal dendrite of an intracellular injected layer III pyramidal neuron of the C40 human cingulate cortex. **(B)** Three-dimensional reconstruction of the complete morphology of each spine shown in (A). **(C)** Estimation of the spine volume values shown in (B) by color codes (blue-white: 0.0–0.8 μm^3^). **(D-I)** Higher magnification images of a dendritic segment shown in A-C to illustrate the three-dimensional triangular mesh (I) obtained for each individual spine. Scale bar in panel I corresponds to: 4.5 μm in panels A-C; 2.5 μm in panels D-F and 1 μm in panels G-I.

**Fig 2:**
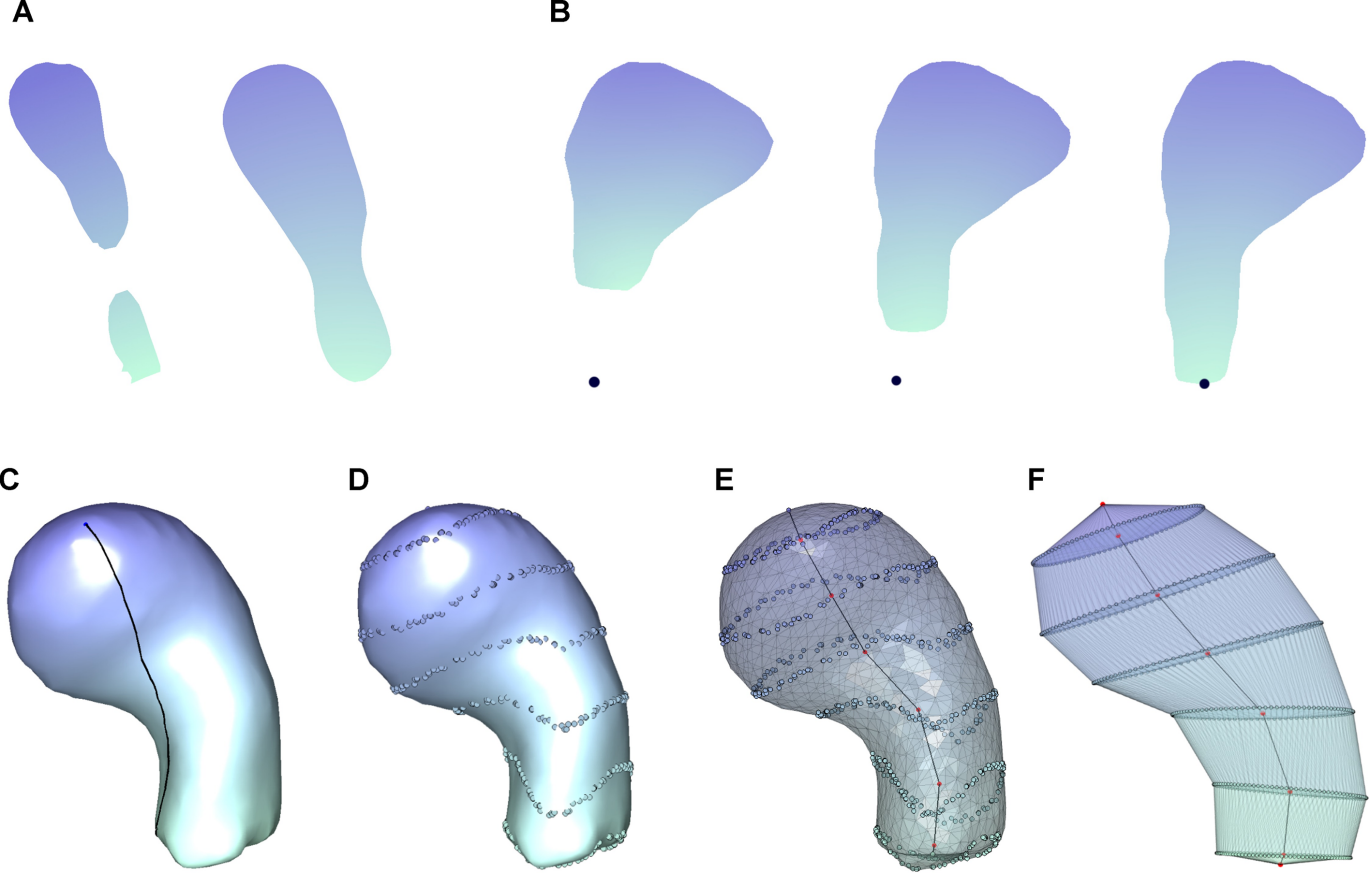
Spine repair process and multiresolutional Reeb graph computation. Spines are colored with a gradient whereby the closest points to the insertion point were colored green and the furthest points were colored purple. **(A)** Example of a fragmented spine. The fragmentation problem is solved by applying the closing morphological operator, and the spine is completely connected. **(B)** Example of the reconstruction of a spine detached from its dendritic shaft. The spine was oriented so as to align the insertion point and its closest vertex with the z-axis. The gap between the spine and the dendritic shaft is filled by means of an iterative process starting from the base of the spine. This resulted in the growth of the missing neck. **(C)** Geodesic distance computation from the insertion point. The black line denotes the shortest path from the insertion point to an arbitrary point on the surface of the spine. **(D)** The domain of the geodesic distance on the surface of the spine was divided into seven regions. **(E)** Regions and segments between curves provide enough information to reconstruct an approximation of the surface. Features extracted from these regions and segments must conform a complete set of spine topology to provide for a proper computer simulation. **(F)** Curves were approximated by the best fitting plane resulting in ellipses that improve the characterization and interpretation of the geometry of the spine. Features were computed on this final 3D representation.

We used a set of 7,916 3D reconstructed individual spines along the apical and basal dendrites of layer III pyramidal neurons in the cingulate cortex of two individuals (aged 40 [C40] and 85 [C85] years) (Fig 1). For each individual spine, a particular threshold was selected to constitute a solid surface that exactly matched the contour of each spine. In many cases, it was necessary to use several surfaces of different intensity thresholds to capture the complete morphology of a spine [15]. In such cases, spines were usually fragmented or detached from their parent dendrite (Fig 2A and 2B) due to the diffraction limitation of confocal microscopy. Therefore, they had to be repaired by means of a novel semi-supervised mesh processing algorithm (see Materials and Methods) which generated a new dataset of corrected spines. Those spines that were extremely fragmented, far removed from the dendrite or significantly deferred from their original shape were discarded. As a result, the original set of 7,916 spines yielded 7,297 (92.18%) spines. The number and percentage of spines after repair by their dendritic compartment and age can be found as S1 Table. For the repair process, the insertion point of each spine was manually marked, approximately at the center of the created spine surface side that was in contact with the dendritic shaft. In those cases where the created spine surface did not reach the dendritic shaft, the insertion point was placed directly on the dendritic shaft where the spine emerged from the shaft, while rotating the image in 3D (Fig 1G). The insertion point was useful for repairing the detached spines and computing a multiresolutional Reeb graph for feature extraction.

The characterization of spines was addressed by dividing the surface of the spine into regions according to a multiresolutional Reeb graph (Fig 2C–2F and Materials and Methods). Thus, regions provided local information on the spine topology while the combination of all regions gave global details of the morphology. Major morphological aspects like length, width, size or curvature were measured for each region to build a set of 36 spine features (see Materials and methods). This set was complemented with 18 features, like growth direction for example. These features were included to achieve an unambiguous representation of the spine morphology. The complete set of 54 features unambiguously describes the position and orientation of all the ellipses that characterize the geometry of a spine. The software to compute the features can be found at https://github.com/ComputationalIntelligenceGroup/3DSpineMFE.

**Fig 3:**
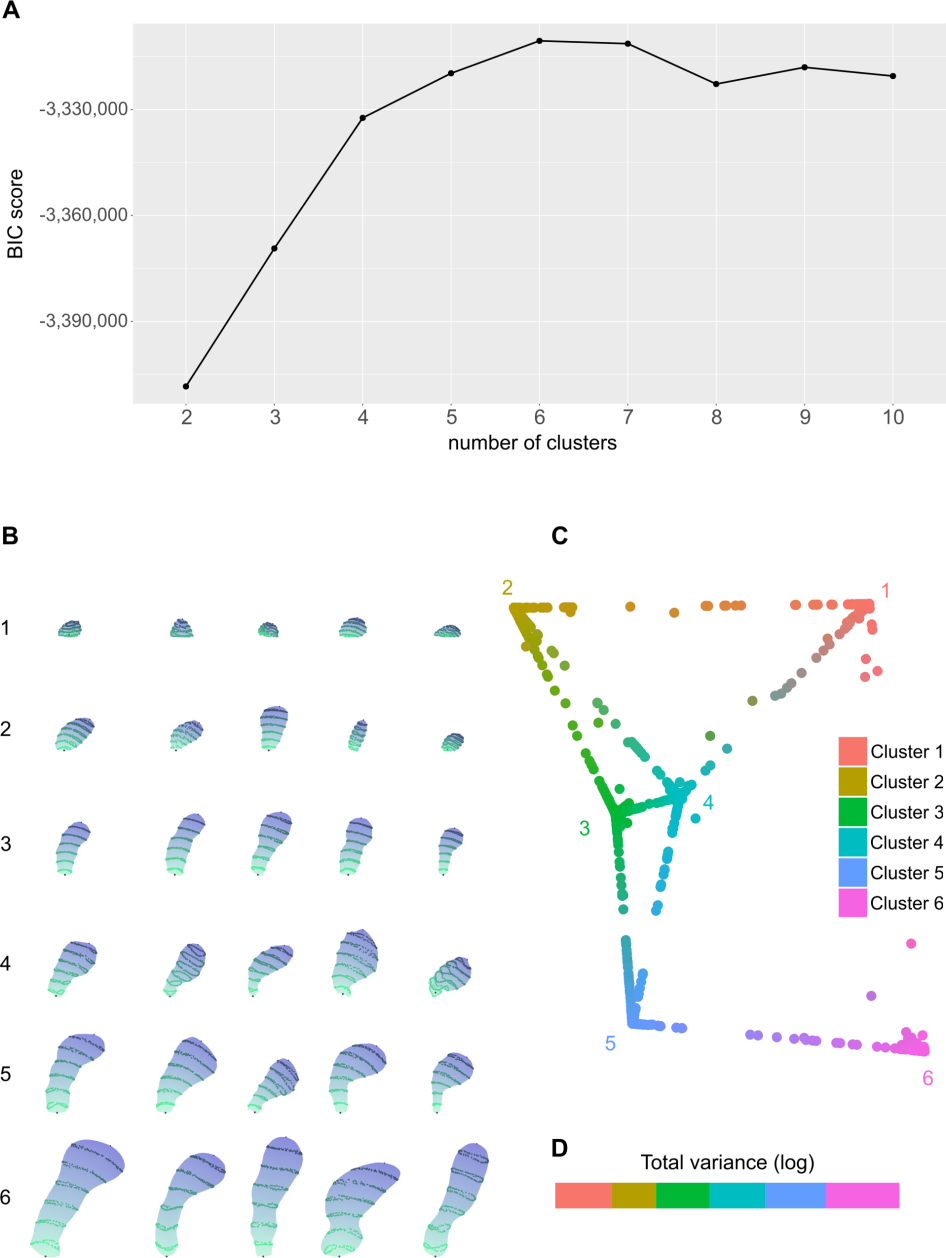
Model-based clustering representation and interpretation. **(A)** Graph showing the resulting BIC values depending on the number of clusters. Results are shown in a range from two to ten clusters. The model that achieved the highest BIC value had six clusters. **(B)** Representative examples of dendritic spines with a p* = 1 (highest membership probability) from the six different clusters. **(C)** 2D projection of the 6D probability distributions representing the membership probability of each spine to each cluster according to classical multidimensional scaling. Spines were colored combining cluster colors according to their probabilities of membership to each cluster. **(D)** The absolute value of the logarithm of the total variance for each cluster, i.e., |log_10_det(**Σ**_*i*_)|, where **Σ**_*i*_ is the variance-covariance matrix of cluster *i*. It is a value that summarizes the heterogeneity of morphologies within a cluster.

To find groups of spines, we applied a model-based clustering approach which assigned spines to six clusters according to the Bayesian information criterion (BIC) (Fig 3A and Materials and Methods). Our approach, based on probabilistic clustering, assigned a probability distribution (*p*_1_,…,*p*_6_) of belonging to each of the six clusters to each spine, where *p*_*i*_ is the probability of belonging to cluster (*p*_*i*_ ∈ [0,1],∑_*i*_*p*_*i*_ = 1). Furthermore, we counted the number of spines whose maximum probability, p* = max{*p*_1_,…,*p*_6_}, was lower than a given threshold (Table 1). We found that the membership probability of most of the spines was greater than 0.99 and clearly belonged to a cluster, whereas a very small number were more scattered and, consequently, their membership was not so clear. Therefore, we can conclude that with this set of features most of spines had very high membership probabilities.

**Table 1:** Number of spines whose maximum probability p* of belonging to a cluster is greater than a threshold.

Prob./Cl.	Cluster 1 (1,025)	Cluster 2 (1,588)	Cluster 3 (1,273)	Cluster 4 (1,454)	Cluster 5 (1,264)	Cluster 6 (693)
0.99	953	1464	1110	1289	1155	648
0.9	995	1558	1229	1404	1226	679
0.8	1008	1575	1248	1425	1244	682
0.7	1013	1581	1257	1441	1254	687
0.6	1016	1585	1268	1448	1260	690
0.5	1025	1588	1273	1454	1264	693

The total number of spines for each cluster is specified between parentheses. Column 1 establishes a threshold probability. Each cell denotes the number of spines that belong to its column cluster with a probability greater than is indicated by its row. For example, 953 spines out of the 1,025 grouped in Cluster 1 had a maximum probability p* greater than 0.99.

### Cluster interpretation and visualization

**Fig 4:**
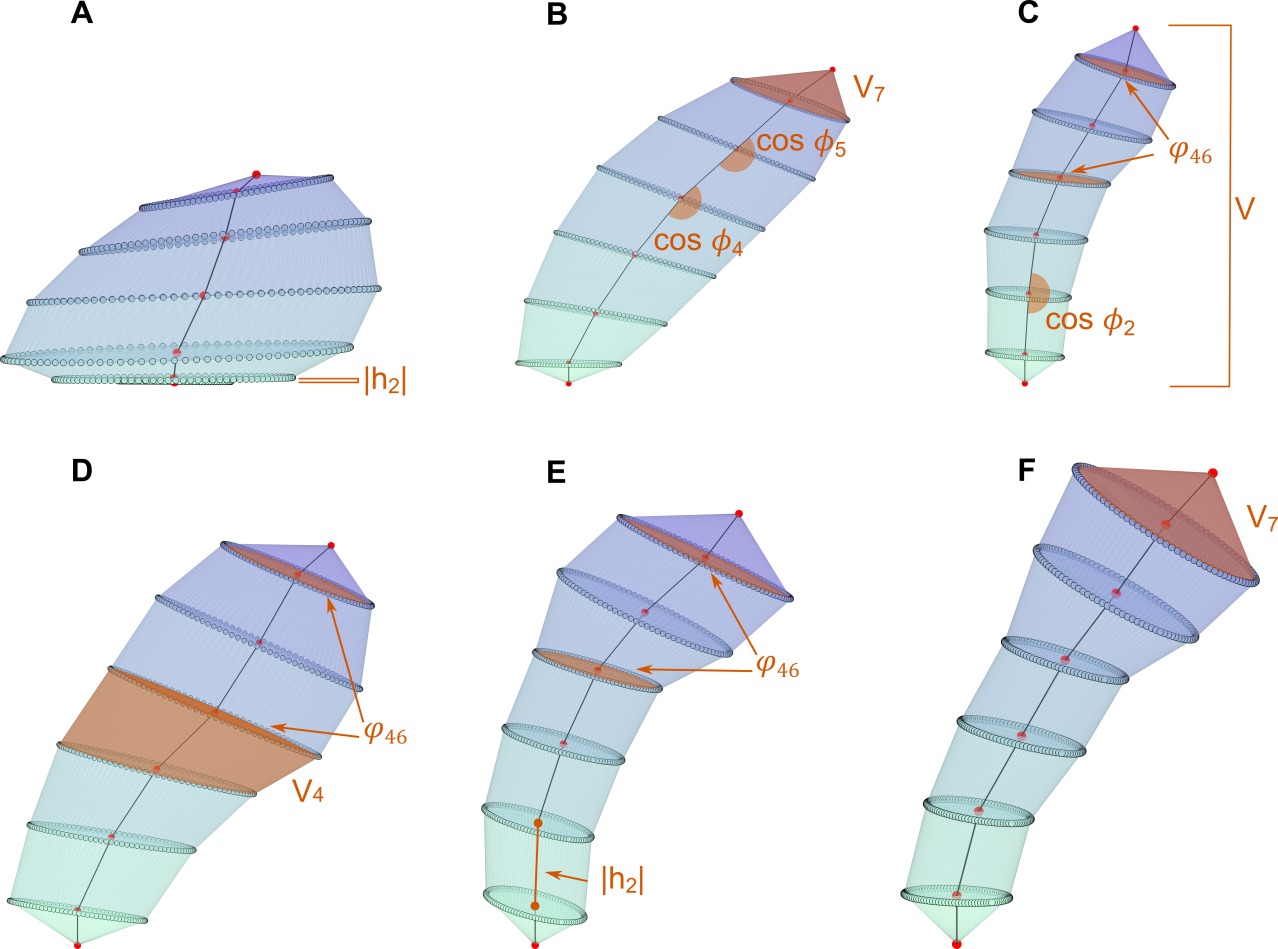
Graphical representations of the main features that characterize each cluster of spines. Representative examples of the spines of each cluster have been rescaled to improve the visualization of their characteristics. The actual proportions between spines are shown in Fig 3B. The correspondences between spines and clusters are: (A) Cluster 1, (B) Cluster 2, (C) Cluster 3, (D) Cluster 4, (E) Cluster 5 and (F) Cluster 6.

To gain a deeper insight into the characterization of each group unveiled by the probabilistic clustering, we identified the most representative features for each cluster. The process was based on the generation of classification rules according to the RIPPER algorithm (see Materials and Methods). Each spine was attributed to its most probable cluster. Then, the RIPPER algorithm generated discriminative rules for each cluster, turning the problem into a binary supervised classification problem which pitched each cluster label against the rest. We forced the algorithm to generate a unique rule in order to improve our understanding of the differences between clusters. However, a single rule cannot be regarded as enough to characterize all the spines within a cluster because it is unable to capture all the relations between the variables defined by the model-based clustering. The result was that each cluster was characterized by only one, two or three observable features (Fig 4). The discriminative rules are available in S1 Text. An example of representative spines of the six clusters is shown in Fig 3B. The rules generated by RIPPER when it comes to classify the spines according to their cluster label, with their accuracy between parentheses, may be summarized as:

- Cluster 1: The height of the spines is extremely low in region 2. (92.94%).
- Cluster 2: Spines with a low curvature across regions 4, 5 and 6 and a small volume in region 7 (80.90%).
- Cluster 3: These spines have a medium-small volume, a low curvature across regions 2 and 3 and the area of their 6th ellipse area may not be more than double or less than half of the area of their 4th ellipse (75.89%).
- Cluster 4: Their volume is high in region 4 and the 6th ellipse has a smaller area than the 4th (82.16%).
- Cluster 5: Groups spines whose height in region 2 is high and whose 6th ellipse has an area that is almost equal to or greater than that of the 4th region (81.95%).
- Cluster 6: Contains the spines with a large volume in region 7 (70.68%).

The diversity of morphologies within a cluster was estimated by computing the total variance for each cluster. Fig 3D shows that Cluster 2 has the lowest total variance, denoting similarity among its spines, whereas variance in Cluster 6 has the highest total variance, suggesting more heterogeneity.

**Fig 5:**
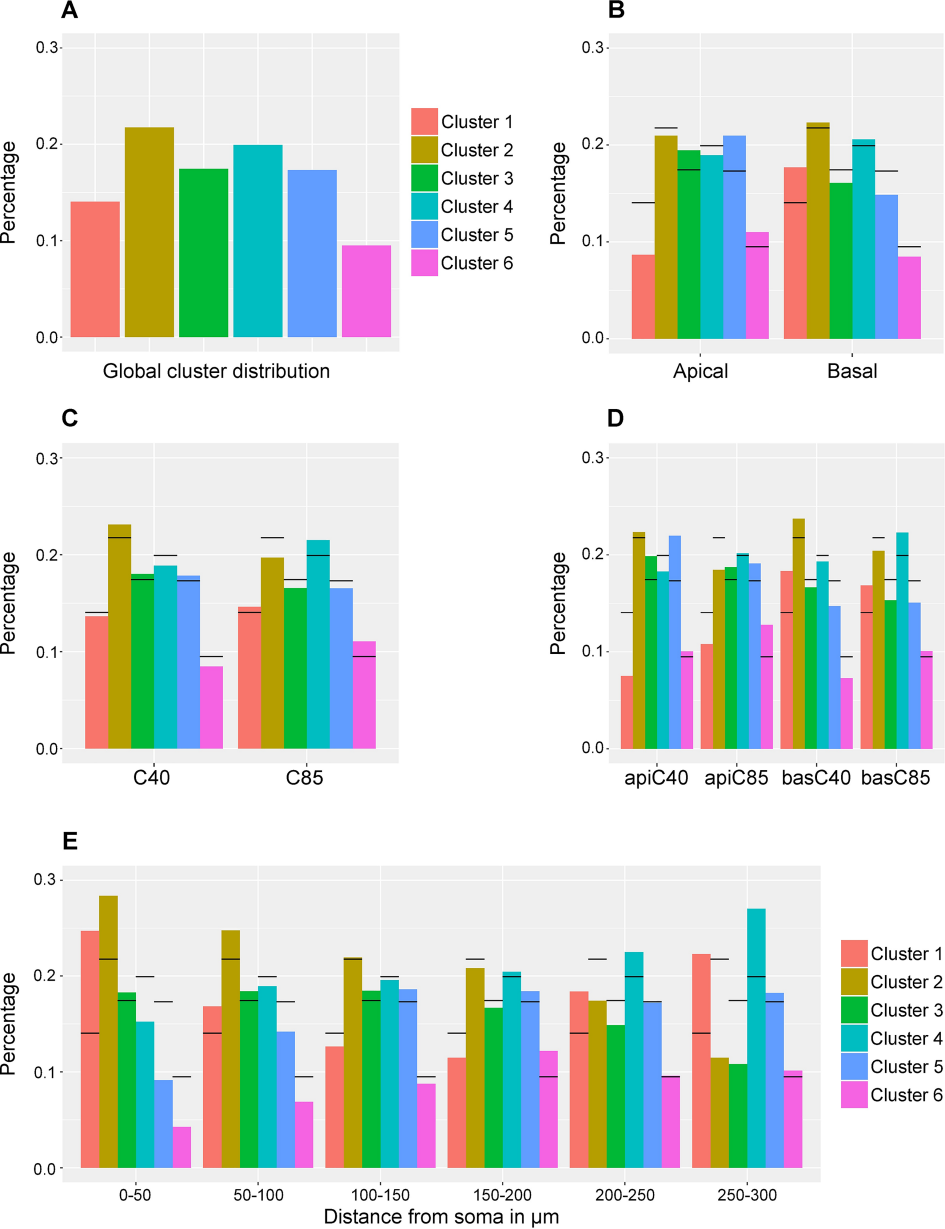
Bar charts showing the distribution of spines by cluster according to maximum probability p*. **(A)** Distribution of spines into the six clusters. **(B)** Relative frequency distribution of clusters for apical (left) and basal (right) spines. **(C)** Relative frequency distribution of clusters for C40 (left) and C85 (right) spines. **(D)** Relative frequency distribution of clusters for the combination of dendritic compartment and age, apical C40 (left end), apical C85 (center left), basal C40 (center right) and basal C85 (right end). Horizontal lines in (B), (C) and (D) denote the heights shown in (A). **(E)** Bar chart showing the distribution of spines belonging to each of the six clusters according to distance from soma. Horizontal lines denote the percentage of spines in each cluster (A). Spines were grouped into intervals of 50 μm to improve visualization.

To improve cluster visualization and interpretation, the distances between the membership probabilities (*p*_1_,…,*p*_6_) of the spines in a 6D space were projected to 2D according to multidimensional scaling (see Fig 3C and Materials and Methods). Spines were colored in line with their probability of belonging to each cluster. Accordingly, “intermediate” spines whose membership probabilities were distributed evenly across several clusters have a mixture of colors. In this representation, we find that most of the points are clearly assigned to a cluster, as suggested by the results reported in Table 1. Clusters 1 and 6 are outstanding examples of a clearly defined cluster, since they are quite isolated and, consequently, easy to discriminate from the other clusters. However, clusters like 3 and 4 are quite closely related. This tallies with the results reported in Table 1, where the clusters identified as being clearly separate had a higher threshold than highly related clusters that needed a lower threshold for all their spines to be crisply assigned. To quantify the distance of the points observed by multidimensional scaling, we measured the overlap between clusters (see Materials and Methods). Note that clearly defined clusters should not overlap. The results reported in S2 Table support the interpretation of multidimensional scaling. By selecting p* of each spine, the spines can be crisply assigned to a unique cluster yielding the distribution shown in Fig 5A. This bar chart represents the percentage of spines that belong to each cluster.

### Distribution of clusters by dendritic compartment, age and distance from soma

To gain a deeper insight, we analyzed how it changes the cluster distribution of the whole population of spines (Fig 5A) when a dendritic compartment (apical/basal), an age (40/85) or a combination of both (Fig 5B–5D) is selected. The study of the cluster distribution of the spines according to their dendritic compartment unveiled that the proportion of spines in Clusters 3, 5 and 6 increase for apical dendrites and diminish for basal dendrites compared with those observed in Fig 5A, whereas the major increment for basal dendrites and decrement for apical dendrites is yielded in Cluster 1. In order to evaluate these differences, we used *χ*^2^ hypothesis testing, that is, we tested whether the cluster distribution is independent of the dendritic compartment (null hypothesis *H*_0_). The hypothesis test returned a p-value lower than 3.80 × 10^−34^ thereby the null hypothesis *H*_0_ was rejected.

The same process as applied for dendritic compartment was repeated for age. Fig 5C shows that Cluster 2 is overrepresented in C40 and Clusters 4 and 6 in C85. On the contrary, the major decreases occur in Cluster 2 in C85 and Clusters 4 and 6 in C40. To test if cluster distribution is independent of age, we tested the hypothesis again. Results rejected the null hypothesis (the p-value was lower than 3.73 × 10^−06^). Furthermore, we run the clustering algorithm for each subject (C40 and C85) to study their distribution independently. As a result, six clusters emerged from C40 spines mostly matching those obtained for the complete population of spines and an additional one of 36 spines that only grouped spines from Clusters 5 and 6. Clustering of C85 spines generated five clusters showing similar results to those achieved for the global population but combining spines from Cluster 2 with Cluster 4 in a unique cluster and tending to include some spines of original Cluster 6 into Cluster 5.

We then tested the cluster distribution and the combination of dendritic compartment and age for independence (Fig 5D). Fig 5D shows that there is an increase of Clusters 3 and 5 for C40 apical dendrites; Clusters 3, 5 and 6 for C85 apical dendrites; Clusters 1 and 2 for C40 basal dendrites and Clusters 1 and 4 for C85 basal dendrites with respect to the distribution observed for the whole population of spines. Additionally, from Fig 5D it can be observed that Clusters 1 and 4 are underrepresented in C40 apical dendrites; Clusters 1 and 2 in C85 apical dendrites; Clusters 5 and 6 in C40 basal dendrites and Clusters 2, 3 and 4 in C85 basal dendrites. The null hypothesis was rejected (p-value ≈ 4.11 × 10^−36^). Hence we can reject independence between cluster distribution and dendritic compartment combined with age.

In spite of the fact that the null hypothesis was rejected for all the above cases, Fig 5B–5D show that the discrepancies in the distributions are confined to only a few clusters and are not evenly spread. With the aim of pinpointing those clusters that exhibit significant differences, each one was analyzed individually. A Pearson’s *χ*^2^ test was performed cluster by cluster to check if the proportion of spines in each individual cluster was independent of the dendritic compartment, age and combination of both. The outcome of the tests is shown in Table 2. Results confirm that only some clusters vary significantly depending on dendritic compartment, age or combination of both and indicate how strongly the hypothesis was rejected for each cluster. An example can be found for age where the null hypothesis was only rejected for Clusters 2, 4 and 6, showing that they are the only clusters whose distribution varies significantly with age.

**Table 2:** Results for Pearson’s *χ*^2^ test checking if the distribution of each cluster is independent of its dendritic compartment, age and combination of both.

	Cluster 1	Cluster 2	Cluster 3	Cluster 4	Cluster 5	Cluster 6
Dendritic compartment	***		**		***	**
Age		*		*		**
Combination	***	*	*	*	***	***

The * symbol denotes that the resulting p-value is lower than 0.05 and the null hypothesis is rejected, ** denotes p-value < 0.001 and *** denotes p-value < 0.0001.

Furthermore, we evaluated the cluster distribution according to the distance from soma (Fig 5E). The number of spines was categorized in 50 μm long sections, from 0 μm (the beginning of the dendrite) to 300 μm. A *χ*^2^ hypothesis test was applied in order to test the independence between cluster distribution and distance from soma. The outcome rejected the null hypothesis *H*_0_ (p-value ≈ 8.00 × 10^−23^). The number of spines assigned to each section is specified in S3 Table. Briefly, Fig 5E shows that there is a predominance of Clusters 1 and 2 at proximal distances (0–50 μm) whereas Clusters 1 and 4 show a higher percentage than expected at longer distances.

### Three-dimensional simulation of spines

**Fig 6:**
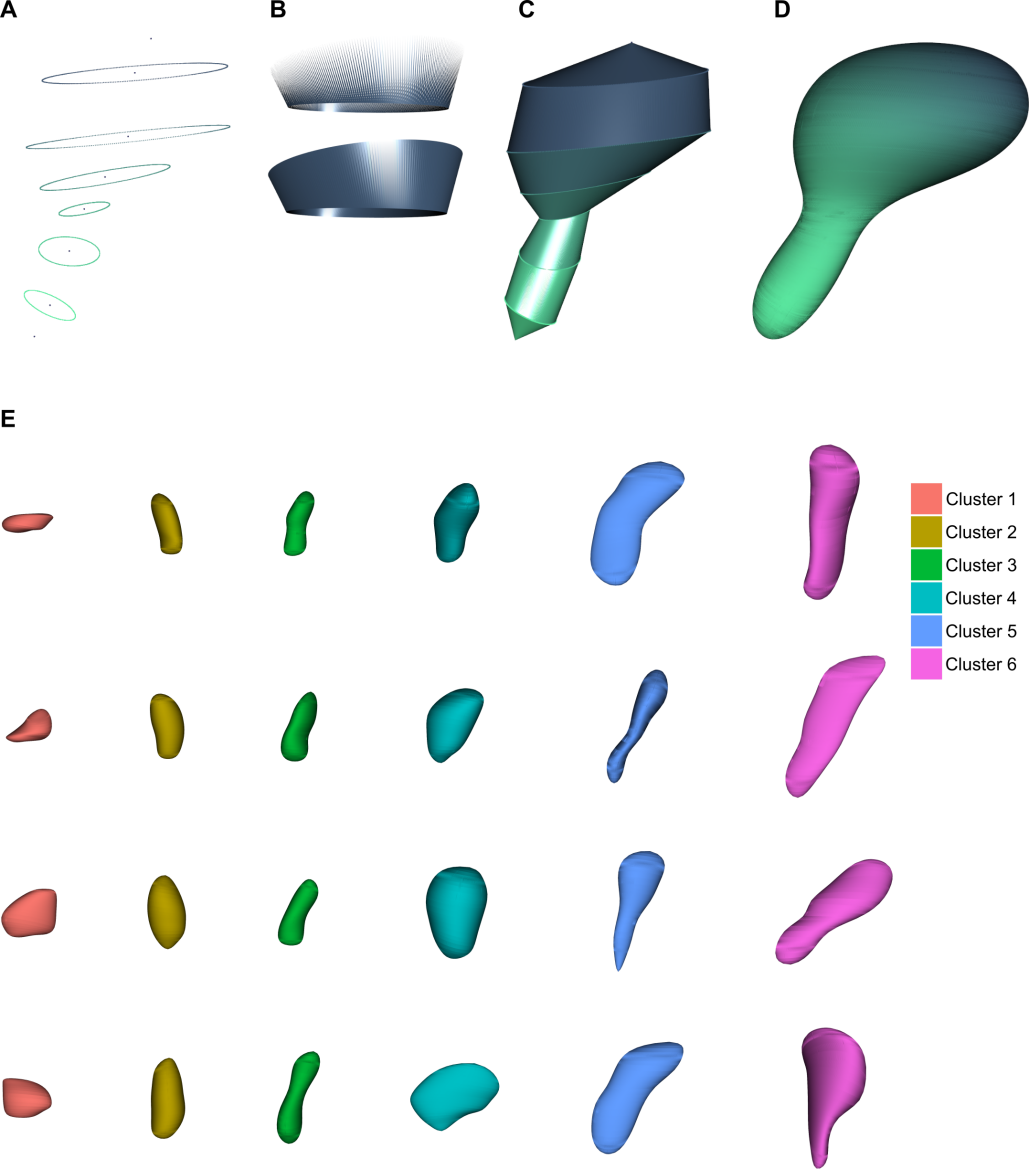
Simulation of 3D dendritic spines. **(A)** Skeleton built from the set of features computed according to the multiresolution Reeb graph. **(B)** Generation of the surface between two ellipses through the triangulation of the region. **(C)** Three-dimensional representation of a spine. Once all the regions of the spine have been triangulated, the spine is a closed mesh used to visualize an artificial spine. **(D)** Improved spine representation. Loop’s subdivision algorithm yields a smoother and more accurate version of the artificial spine. **(E)** Examples of simulated spines for each cluster.

Model-based clustering describes the probability distributions governing each cluster. Given a cluster, a spine is simulated sampling the values for the 54 features from its probability distribution (see Materials and Methods). This set of features unambiguously specifies the position and orientation of ellipses that define the skeleton of a simulated spine (Fig 6A). The simulated spine is represented in 3D by surfacing the skeleton (Fig 6B). However, simulated spines have an artificial appearance because the regions delimited by the ellipses are clearly distinguishable between them (Fig 6C). A more accurate morphology for the simulated spine is generated by smoothing the surface (Fig 6D). Examples of simulations of each cluster can be found in Fig 6E. R code, model and dataset to perform clustering and simulation of dendritic spines can be downloaded from https://github.com/sergioluengosanchez/spineSimulation.

To be useful for future research, simulated spines must be geometrically equivalent to real spines. Thus, simulated and real must be indistinguishable. To test for equivalence, we state a supervised classification problem within each cluster, where the possible labels are “simulated” vs. “real”. Hence, if both groups were indistinguishable, a classifier would perform badly, having a classification accuracy of around 50%. As a result we found that both groups of spines are almost indistinguishable (accuracy being around 60%), with the exception of cluster 1 (80%), where the size of simulated spines is usually somewhat larger than real spines.

## Discussion

This study illustrates the geometrical clustering results from over 7,000 complete manual 3D reconstructions of human cortical pyramidal neuron spines. Specifically, we uncovered six different classes of human spines according to a particular set of features. Additionally, we found that particular clusters were predominant in different dendritic compartments, ages and distances from soma. Furthermore, we created 3D virtual representations of spines that matched the morphological definitions of each cluster. To the best of our knowledge, this is the first time that such a large dataset of individual manually 3D reconstructed spines from identified human pyramidal neurons is used to automatically generate objective morphological clusters with a probabilistic model.

Technically, serial electron microscopy is the technique of choice to obtain highly accurate measurements of the dendritic spine structure. However, it is very time-consuming and difficult, which makes it challenging to obtain large numbers of measurements. Even using high throughput 3D reconstruction of identified dendritic spines by means of automatic electron microscope techniques such as FIB/SEM technology (combined use of focused ion beam milling [FIB] and scanning electron microscopy [SEM]), the number of reconstructed spines is relatively low. For example, FIB/SEM technology has permitted the full 3D reconstruction of up to 248 spines and their synaptic inputs in the adult-generated granule cells in mice [22], which represents a major achievement in the field. Light microscopic techniques, although limited by the lower level of resolution, remain the method of choice to obtain large-scale spatial information regarding the number and distribution of dendritic spines along the dendrites (in the order of several thousands of spines). Nevertheless, light microscopic studies normally estimate dendritic spine volumes from measurement of the spine head volumes, whereas spine necks are usually not included, due to the lack of software tools to reconstruct these structures accurately and because some of the spine necks have spatial dimensions of around 50–200 nm and, therefore, are not resolvable by confocal microscopy. Moreover, as discussed by Tønnesen and Nägerl [14], image projection artifacts and limited spatial resolution mask short spine necks, leading to the false identification of stubby spines. In addition, it is difficult to distinguish the border between the head and the neck in many cases. Thus, in the present study, we used 3D reconstructed dendritic spine morphology using commercially available module software (Imaris surface), which allowed us to create our own protocol to accurately represent the morphology of the spine within the limits of light microscopy (see [15]).

Model-based clustering methods used in this study yield six clusters based on their BIC value (see Fig 3A). This criterion resulted in high cluster membership probabilities for this set of features. These included measurements of major morphological aspects like length, width or size of the spine but also other aspects such as curvature. Thus, these and previous results [3,15], where only their volume and length were measured, are not comparable.

Interestingly, we observed that there are particular clusters of spines that are proportionally highly represented in a particular dendritic compartment/age combination. Specifically, basal dendrites contained a higher proportion of the small Cluster 1 spines (Fig 3B), whereas apical dendrites contained a higher proportion of the medium/large Clusters 3, 5 and 6 spines. These differences would imply that their functional properties should be expected to be different in the two dendritic compartments [2]. Regarding individuals, Cluster 2 spines accounted for a higher percentage in the younger individual, whereas Clusters 4 and 6 of bigger spines had higher values than the mean percentage in the older individual. Since small spines have been reported to be preferential sites for long-term potentiation induction and large spines might represent physical traces of long-term memory [9,13], the results suggest that the younger individual has a higher potential for plasticity than in the aged case. The dendritic compartment/age combination results also agreed with our previously reported study [15] that found that apical dendrites have longer spines than basal dendrites, and younger basal dendrites are significantly smaller than aged basal dendrites. For instance, small and short spines of aged basal dendrites and long spines of apical dendrites were lost. Regarding the distance from soma, there is a higher predominance of the small Clusters 1 and 2 spines than expected at proximal distances (0–50μm) and the small Cluster 1 spines at distal distances. Also, distal distances showed a higher percentage of the medium-sized Cluster 4 spines than expected. Since variations in spine geometry reflect different functional properties of the spine, this particular distribution of spines might be related to the morphofunctional compartmentalization of the dendrites along the length of the dendritic pyramidal neurons. For example, it has been reported that different domains of the basal dendritic arbors of pyramidal cells have different properties with respect to afferent connectivity, plasticity and integration rules [15,19,23–26]. Thus, these results may be a reflection of a functional dendritic organization based on spine geometry.

Using the technique of model-based clustering described in this study, we were able to simulate accurate spines from human pyramidal neurons. This is important for three main reasons. First, it is not necessary to store large volumes of data because all the information is summarized in the mathematical model. Second, spines are known to be dynamic structures (see [27] for a recent review), and changes in spine morphology have important functional implications potentially affecting not only the storage and integration of excitatory inputs in pyramidal neurons but also mediating evoked and experience-dependent synaptic plasticity. This, in turn, has major repercussions on cognition and memory [13,28–32]. Thus, it is necessary to link the structural data with theoretical studies and physiological data on spines in order to interpret and make the geometrical data on spines more meaningful. Functional modeling of spines is commonly carried out according to their values of surface area, spine maximum diameter, spine neck diameter, spine length, and spine neck length. Since each cluster contains a spine population with a range of morphological features, it is necessary to model all of these morphological variations within each cluster in order to compare the possible functional differences between the clusters found in the present study. Third, one of the major goals in neuroscience is to simulate human brain neuronal circuitry based on data-driven models because ethical limitations prevent all of the necessary datasets from being acquired directly from human brains. Therefore, the implementation of this mathematical model of spines of human pyramidal cells in current models of pyramidal neurons is a potentially useful tool for translating neuronal circuitry components from experimental animals to human brain circuits. The simulation of the spines in this study represents a mathematical model that could be implemented in pyramidal cell models [33] in order to present the data in a form that can be used to reason, make predictions and suggest new hypotheses of the functional organization of the pyramidal neurons. Finally, spine heads and necks of human pyramidal cells are significantly larger in terms of their area and longer, respectively, than mouse spines [34]. Therefore, it would be interesting to compare human and non-human spines using the present model-based clustering to ascertain whether the clusters that appear are the same or different in other species, or whether there are differences between different cortical areas.

## Materials and methods

### Ethics statement

Brain samples were obtained from the Institute of Neuropathology Brain Bank, a branch of the HUB-ICO-IDIBELL Biobank and member of the Spanish Biobank network (RETIC Biobank) of the Institute of Health Carlos III, following the guidelines of Spanish legislation (real Decreto 1716/2011) and the approval of the local ethics committee, and in accordance with recently published criteria for sample quality (PMID: 25113170).

### Tissue preparation

A set of 7,916 individually 3D reconstructed spines from layer III pyramidal neurons from the cingular cortex of two human males (aged 40 [C40] and 85 [C85]) were used for analyses. These cases were used as controls in a previous study unrelated to the present investigation that was dealing with Alzheimer’s disease [35]. The cause of death was traffic accident (case C40) and pneumonia plus interstitial pneumonitis (aged case, C85). The tissue (kindly supplied by Dr I. Ferrer, Instituto de Neuropatología, Servicio de Anatomía Patológica, IDIBELL-Hospital Universitario de Bellvitge, Barcelona, Spain) was obtained at autopsy (2–3 h post-mortem). The brains were immediately immersed in cold 4% paraformaldehyde in 0.1 M phosphate buffer, pH 7.4 (PB) and sectioned into 1.5-cm-thick coronal slices. Small blocks of the cortex (15 × 10 × 10 mm) were then transferred to a second solution of 4% paraformaldehyde in PB for 24 h at 4°C. After fixation, vibratome sections (250 μm) from the anterior cingular gyri (Brodmann's area 24;[36]) were obtained with a Vibratome and labelled with 4,6 diamino-2-phenylindole (DAPI; Sigma, St Louis, MO) to identify cell bodies. Pyramidal cells were then individually injected with Lucifer Yellow (LY; 8% in 0.1 M Tris buffer, pH 7.4), in cytoarchitectonically identified layer III of the anterior cingular gyrus. LY was applied to each injected cell by continuous current until the distal tips of each cell fluoresced brightly, indicating that the dendrites were completely filled and ensuring that the fluorescence did not diminish at a distance from the soma (for a detailed methodology of the cell injections, see [37–39]). Apical and basal dendrites were then scanned at high magnification by confocal microscopy and reconstructed in three dimensions using a methodology previously described in detail [15]. Sections were imaged with a Leica TCS 4D confocal scanning laser attached to a Leitz DMIRB fluorescence microscope. Fluorescent labeling profiles were imaged, using an excitation wavelength of 491 nm to visualize Alexa fluor 488. Consecutive stacks of images (3 ± 0.6 stacks per dendrite; 52 ± 17 images) were acquired at high magnification (×63 glycerol; voxel size, 0.075 × 0.075 × 0.28 μm^3^) to capture the full dendritic depth, length, and width of basal dendrites, each originating from a different pyramidal neuron (10 per case). The voxel size was calculated to acquire images at the highest resolution possible for the microscope which made it possible to capture the traditional diffraction limits of fluorescence microscopy (approximately 200 nanometers). Regarding apical dendrites, the main apical dendrite was scanned, at a distance of 100 μm from the soma up to 200 μm (8 dendrites per case). Thus, no apical dendritic tufts were included in the analyses. As a result, a dataset containing 7,916 3D reconstructed individual spines along the apical and basal dendrites was built (Fig 1).

### Repairing spines

We addressed the task of repairing spines by means of a semi-automatic mesh processing algorithm (Fig 2A). The procedure starts by identifying fragmented spines. A spine is fragmented if there is no path between every pair of vertices on the surface of the 3D mesh, and all the vertices belong to a closed mesh. If this is the case, fragmentation is repaired by applying a closing morphological operator to each spine individually. This operator requires a binary image as input, and therefore 3D meshes are voxelized [40]. As a result of applying the closing operator to each voxel of the volumetric spine using a sphere as a structuring element, fragments are joined to form a single body. The marching cubes algorithm [41] recovers the mesh representation from the volumetric image of the repaired spine.

The repair process was continued by connecting spines to dendrites by means of spine path reconstruction (Fig 2B). Several points were created to attach the spine to the dendrite, using the measurement point tool in Imaris software. These are considered to be the spine insertion points. Spine reconstruction was applied to any spines whose insertion point was not on the surface of the mesh. This step in the repair process consists of filling the gap between the closest vertex of the spine to the insertion point and the insertion point according to an iterative process that grew the missing base of the spine. Specifically, each detached spine was oriented so that both points bounding the gap were aligned with the z-axis. Then, the mesh of each spine was voxelized. Each voxel slice perpendicular to the z-axis between the spine and the insertion point was filled with the result of applying a 2D Gaussian filter to the slice immediately above. The mesh representation of the completely repaired spine was recovered from the volumetric representation by the marching cubes algorithm. Finally, we smoothed the triangular mesh with a curvature flow technique [42]. As result of this process a new dataset of corrected spines is obtained.

### Feature extraction

Given 3D meshes representing the surface of the spines, our goal was to extract a set of morphological features providing enough information to reconstruct an approximation of their original shapes. Our work was partially inspired by the concept of multiresolutional Reeb graph (MRG) [43] and its particular implementation in [44], a technique that constructs a graph from a 3D geometric model to describe its topology (Fig 2C–2F). This approach partitions a triangular mesh into regions based on the value of a function *μ*(⋅). This function should preferably be the geodesic distance, i.e., the shortest path between two points of the mesh along the surface because it is invariant to translation and rotation and is robust against mesh simplification and subdivision. We computed geodesic distance from the insertion point of the spine to each vertex of the mesh (Fig 2C). The domain of *μ*(⋅) was divided into K = 7 equal length intervals, where *r*_*i*_ indicates the beginning and the end of each region such that $r_{0}=\left[0,\frac{1}{K}\alpha \right],r_{1}=\left(\frac{1}{K}\alpha ,\frac{2}{K}\alpha \right],\ldots ,r_{K-1}=\left(\frac{K-1}{K}\alpha ,\alpha \right]$, where *α* is max *μ*(⋅). This means that each of the vertices in the triangular mesh was allocated to a particular region depending on its evaluation function *μ*(⋅) (Fig 2D–2E). At each region *i*, the curves defining the top and bottom bounds were assumed to be ellipses contained in the best fitting plane computed using principal component analysis. We denote *T*_*i*_ and *B*_*i*_ the top and the bottom ellipses of each region *i* respectively. Thus, each region provided a local description of the morphology while the combination of the information of all regions represented a global characterization of the spine. Representing a spine as a set of ellipses allows us to capture its most relevant morphological aspects while spurious details are avoided.

**Fig 7:**
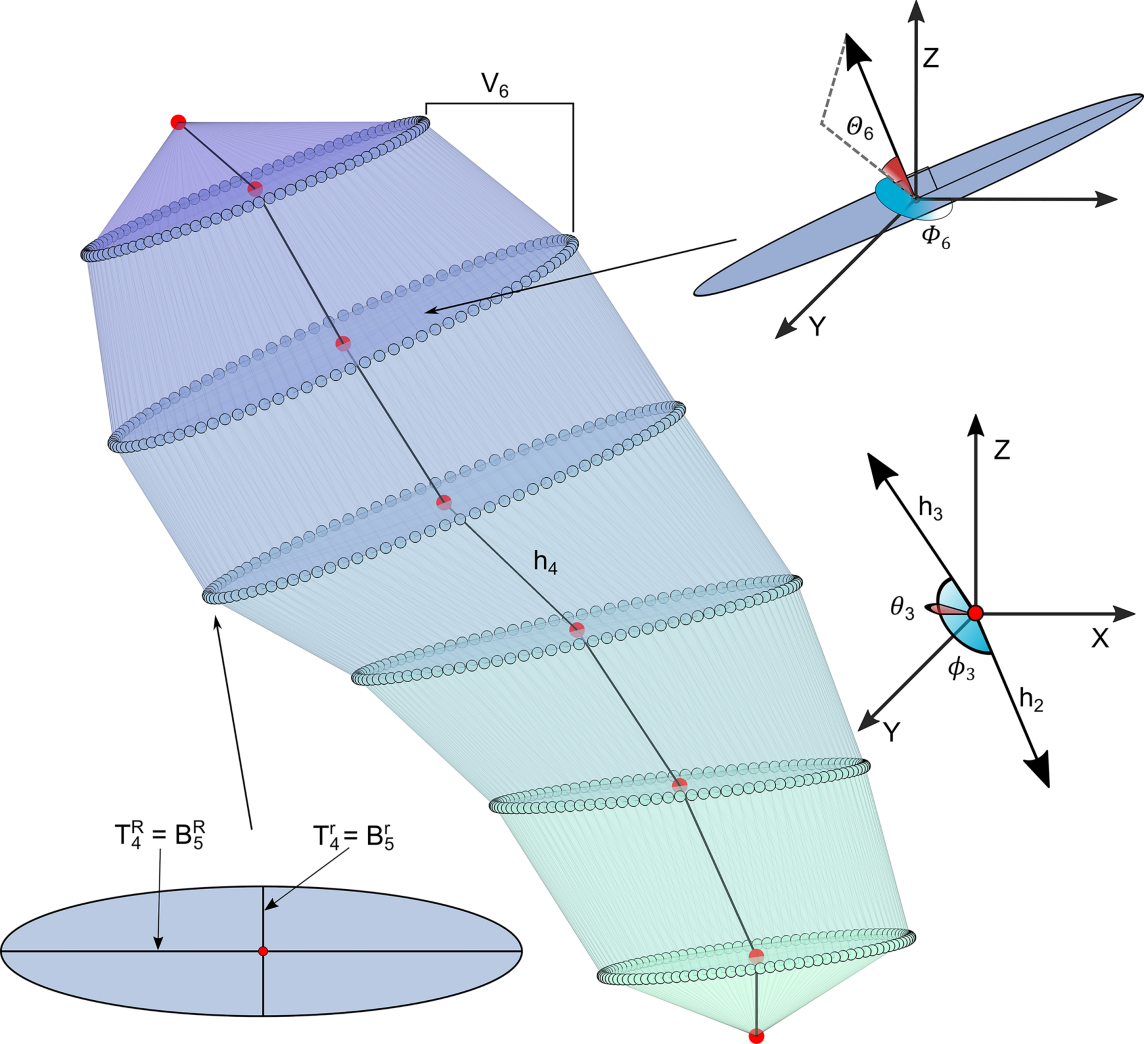
Spine features description. An ellipse is defined by its centroid, major axis ($T_{i-1}^{R}=B_{i}^{R}$) and minor axis ($T_{i-1}^{r}=B_{i}^{r}$). These points are connected by vectors ***h***_*i*_ whose length is |***h***_*i*_|. From vectors ***h***_*i*_ and ***h***_*i***−1**_, ***θ***_***i***_ and ***ϕ***_***i***_ are obtained. ***Θ***_***i***_ and ***Φ***_***i***_ are the ellipse directions of the spine.

The proposed set of 54 features must unambiguously describe the placement of the ellipses. To achieve this, at each region *i* a set of features was computed according to their ellipses *T*_*i*_ and *B*_*i*_. Since the surface was required to be continuous coherence constraints were imposed on adjacent regions: $\forall i,1<i<K+1,{B_{i}^{R}=T}_{i-1}^{R},{B_{i}^{r}=T}_{i-1}^{r}$. Thus, to satisfy the previous condition the following features were considered to characterize the spine (Fig 7):

Height (|***h***_*i*_|)): This variable measures the length of the vector ***h***_*i*_ between the centroids of two consecutive ellipses. The higher the value of this variable, the longer the spine in that region.

Length of major axis of ellipse $\left(B_{i}^{R}\right)$: Low values mean that spine is thin around $B_{i}^{R}$.

Length of minor axis of ellipse $\left(B_{i}^{r}\right)$: It gives information about the squishiness of the spine when it is compared with $B_{i}^{R}$. If $B_{i}^{R}$ and $B_{i}^{r}$ have the same values the ellipse is in fact a circle while when $B_{i}^{r}$ gets smaller the ellipse becomes more squished.

Ratio between sections (*φ*_*ij*_): It is the ratio between the area of the ellipses *j* and *i*, i.e., $\varphi_{ij}=\frac{\pi B_{j}^{R}B_{j}^{r}}{\pi B_{i}^{R}B_{i}^{r}}$. If it is higher than 1 it means that ellipse *j* is bigger than ellipse *i*. When values are between 0 and 1 it means that ellipse *i* is bigger than ellipse *j*. It can be interpreted as the widening or narrowing along the spine. We compute *φ*_24_, *φ*_26_ and *φ*_46_.

Growing direction of the spine: The vector between ellipse centroids ***h***_*i*_ defines a direction which can be expressed in spherical coordinates, i.e., an azimuth angle *ϕ*_*i*_ and an elevation angle *θ*_*i*_.

- cos(*ϕ*_*i*_): Cosine of the azimuth or azimuthal angle, obtained as the angle between the vectors defined by three consecutive ellipses. The cosine is computed from the dot product: $\cos\left(\phi_{i}\right)=\frac{h_{i}\cdot h_{i+1}}{\left|h_{i}||h_{i+1}\right|}$. It measures the curvature of the spine.
- *θ*_*i*_: The polar angle, also called colatitude in the spherical coordinate system. It is needed for simulation.

Ellipse direction: It is the direction of the perpendicular vector to ellipse *B*_*i*_. It is obtained from $\frac{B_{i}^{R}}{\left|B_{i}^{R}\right|}\times \frac{B_{i}^{r}}{\left|B_{i}^{r}\right|}$ (vectorial product). It is expressed in spherical coordinates as:

- *Θ*_*i*_: The polar angle or colatitude in spherical coordinate system. It is the inclination of the vector perpendicular to the ellipse with respect to $\vec{Z}$ axis. If it is 0 then the spine grows horizontally at that point. When it is $\frac{\pi }{2}$, it means that the spine grows completely vertical at that point. It is needed for simulation.
- *Φ*_*i*_: The azimuth or azimuthal angle. It indicates if the spine is growing to the right, left, forward or backward as it was previously explained for the growing direction but in this case it is computed for the perpendicular vector to the ellipse. It is needed for simulation.

Volume (V): It is the total volume of the spine.

Volume of each region (*V*_*i*_): It an approximation of the volume between two consecutive ellipses. It is computed from the convex hull of *T*_*i*_ and *B*_*i*_.

By generating a surface between each pair of ellipses, we get an approximation of the shape of the spine (Fig 6B and 6C). Surfaces between regions can be computed by the method that we propose in the spine simulation section under Materials and Methods.

### Model-based clustering

Model-based clustering [45] is a probabilistic approach that assumes that data were generated by a statistical model. Its goal is to recover that model from the observed data. Finite mixture models provide a formal setting for model-based clustering. In finite mixture models, each cluster is represented by a probability distribution. The linear superposition of such distributions generates the finite mixture *M*. The fit of the model to the data depends on a set of parameters that are usually optimized by means of maximum-likelihood estimation. This estimation method finds the set of parameters ***θ*** that maximize the observed data likelihood, i.e., max_***θ***_ *f*(***x***_1_,…,***x***_*N*_|***θ***), where ***x***_1_,…,***x***_*N*_ are a data sample of size *N*. Then, we assume that the vector of features describing the spines is distributed according to a Gaussian mixture, as it can approximate any multivariate density given enough components [46]. Thus, the density is

$$f(x_{1},\ldots ,x_{N}|\theta )=\sum_{c=1}^{C}\pi_{c}N(x|\mu_{c},\Sigma_{c}),$$

where N denotes a multivariate normal distribution with prior probability *π*_*c*_, mean vector ***μ***_*c*_ and variance-covariance matrix **Σ**_*c*_, *C* is the total number of clusters and each cluster is denoted by *c*. Thus, the goal is to get the values for the set of parameters ***θ*** = {*π*_*c*_,***μ***_*c*_,**Σ**_*c*_}_*c*_ that maximize the likelihood. This was approximated using an iterative two-step procedure called expectation-maximization algorithm [47]. To choose the most suitable number of clusters, we computed the Bayesian information criterion (BIC) score [48] for different values of *C*. BIC is a measure that adds a penalty to the log-likelihood of the model based on the number of model parameters. Therefore, it is used to select the best parameterization and number of clusters by trying to avoid the selection of overly complex models.

After the clustering process, each spine has a certain probability of belonging to each cluster (“soft” clustering). We used mclust, a contributed R package [49], for model-based clustering and density estimation.

### Clustering interpretation tools

In order to shed light on the features that characterize each cluster, we generated classification rules according to the RIPPER algorithm [50]. The spines were crisply assigned to a unique cluster by selecting the most probable cluster for each spine. Then, RIPPER compared each cluster against the others, generating discriminative rules. SMOTE [51] was applied as a pre-processing step before running RIPPER to avoid bias and deal with the unbalanced distribution of instances arising from data splitting (one cluster versus the rest). SMOTE is a technique for adjusting the class distribution so that the set of observations of the least represented class is resampled. We also forced RIPPER to select a unique rule to improve the interpretability of each cluster, highlighting its most discriminative features. We used the RIPPER implementation included in the collection of algorithms of Weka, a software for machine learning tasks [52].

To make the clustering results graphically interpretable, we applied multidimensional scaling to represent the distance of spines to clusters according to their membership probability (Fig 3C). To achieve this goal, distances between each pair of multivariate Gaussians defined by the clusters were calculated according to the Bhattacharyya distance [53]. Based on this measure, we were then able to project the above distances, originally in a 6D space, onto a 2D space using multidimensional scaling [54]. Thus, spines were placed in this space in proportion to the probability of their belonging to each cluster.

Clustering aims to group similar instances and separate dissimilar instances. Therefore, method performance depends on whether the clusters overlap with each other. Non overlapping clusters are easily discovered. However, clustering algorithms have trouble separating overlapped clusters because instances cannot be clearly assigned to clusters. Hence, overlapping was understood according to [55,56] as the probability of misclassifying an instance from cluster *i* in a cluster *j*. Thus, the probability *ω*_*j*|*i*_ of misclassifying an instance of the *i*-*th* component to the *j*-*th* component was computed as

$$\omega_{j|i}=P[\pi_{i}N(x|\mu_{i},\Sigma_{i})<\pi_{j}N(x|\mu_{j},\Sigma_{j})|x∼N(\mu_{i},\Sigma_{i})].$$

### Spine simulation

The simulation process aimed at achieving accurate 3D representations of spines generated by the computer. This process is divided into two main phases.

First, we sampled new instances from the mixture model of multivariate Gaussians. As a result of sampling, we got a dataset where each instance consisted of a vector with 54 feature values defined by a multiresolution Reeb graph.

Second, we generated a 3D representation for each instance. From the set of features of a sampled spine, we built a skeleton composed of the ellipses establishing the beginning and end of regions (Fig 6A). Because all the ellipses had the same number of points, each pair of consecutive ellipses was easily triangulated to obtain a closed mesh (Fig 6B and 6C). Although this mesh is a 3D spine, ellipses are clearly distinguishable. We improved this result by smoothing the surface with the Loop’s subdivision algorithm [57]. Thus, we obtained a more accurate 3D representation of the spine (Fig 6D).

To objectively validate the realism of the simulated spines, we used a binary classifier, specifically the RIPPER algorithm. First, for each cluster, we sampled from the probability distribution of each cluster the same number of simulated spines as real spines are. Second, we combined these with real spines to generate a dataset for each cluster. Third, we applied the RIPPER algorithm with ten-fold cross-validation [58] over the datasets to discriminate between real and simulated spines. This process yields classifier accuracy, which can be regarded as the degree of realism.

## Supporting information

S1 Table Number and percentage of spines after repair by their dendritic compartment and age. (DOCX)

S2 Table This table reports the probability of misclassifying a spine from cluster *i* in cluster *j*. The probability of classifying a spine from Cluster 3 in Cluster 4 is 1.69e-05. These values are interpreted as a measure of the overlap between clusters. Spines that are not clearly assigned to a cluster are placed between clusters that overlap. This matches the relations between clusters observed in the multidimensional scaling representation.
(DOCX)

S3 Table Number of dendritic spines as a function of their distance from the soma. The spines were ascribed to sections of 50 μm long, from 0 μm (beginning of the dendrite) to 300μm.
(DOCX)

S1 Text Supplementary information. Plain discriminative rules and summary statistics of the features.
(DOCX)
