# Supplementary material for: 3D morphology-based clustering and simulation of human pyramidal cell dendritic spines
Source: PLoS Comput Biol. 2018 Jun 13;14(6):e1006221. doi: 10.1371/journal.pcbi.1006221 (PMC6060563; doi:10.1371/journal.pcbi.1006221)
Supplement: S1 Table — (DOCX) [file pcbi.1006221.s001.docx]

**S1 Table**: **Number and percentage of spines after repair by their dendritic compartment and age.**

|  | C40 | C85 | Sum |
| --- | --- | --- | --- |
| Apical | 1,893 (26%) | 1,057 (14%) | 2,950 (40%) |
| Basal | 2,500 (34%) | 1,847 (26%) | 4,347 (60%) |
| Sum | 4,393 (60%) | 2,904 (40%) | 7,297 (100%) |
