# Supplementary material for: 3D morphology-based clustering and simulation of human pyramidal cell dendritic spines
Source: PLoS Comput Biol. 2018 Jun 13;14(6):e1006221. doi: 10.1371/journal.pcbi.1006221 (PMC6060563; doi:10.1371/journal.pcbi.1006221)
Supplement: S2 Table — The probability of classifying a spine from Cluster 3 in Cluster 4 is 1.69e-05. These values are interpreted as a measure of the overlap between clusters. Spines that are not clearly assigned to a cluster are placed between clusters that overlap. This matches the relations between clusters observed in the multidimensional scaling representation. (DOCX) [file pcbi.1006221.s002.docx]

**S2 Table: This table reports the probability of misclassifying a spine from cluster** $\boldsymbol{i}$ **in cluster** $\boldsymbol{j}$**.**

|  | Cluster 1 | Cluster 2 | Cluster 3 | Cluster 4 | Cluster 5 | Cluster 6 |
| --- | --- | --- | --- | --- | --- | --- |
| Cluster 1 | 1 | 2.26e-07 | 2.09e-07 | 1.05e-06 | 1.02e-10 | 1.75e-11 |
| Cluster 2 | 6.94e-08 | 1 | 8.62e-06 | 8.29e-06 | 2.36e-10 | 0 |
| Cluster 3 | 1.53e-07 | 2.35e-05 | 1 | 1.69e-05 | 2.00e-05 | 1.20e-10 |
| Cluster 4 | 7.79e-07 | 2.00e-05 | 1.53e-05 | 1 | 4.54e-06 | 1.35e-09 |
| Cluster 5 | 2.08e-10 | 7.96e-10 | 4.08e-05 | 1.06e-05 | 1 | 5.88e-06 |
| Cluster 6 | 1.64e-10 | 0 | 2.69e-10 | 5.79e-09 | 1.63e-05 | 1 |

The probability of classifying a spine from Cluster 3 in Cluster 4 is 1.69e-05. These values are interpreted as a measure of the overlap between clusters. Spines that are not clearly assigned to a cluster are placed between clusters that overlap. This matches the relations between clusters observed in the multidimensional scaling representation.
