# Supplementary material for: 3D morphology-based clustering and simulation of human pyramidal cell dendritic spines
Source: PLoS Comput Biol. 2018 Jun 13;14(6):e1006221. doi: 10.1371/journal.pcbi.1006221 (PMC6060563; doi:10.1371/journal.pcbi.1006221)
Supplement: S3 Table — The spines were ascribed to sections of 50 μm long, from 0 μm (beginning of the dendrite) to 300μm. (DOCX) [file pcbi.1006221.s003.docx]

**S3 Table**: **Number of dendritic spines as a function of their distance from the soma.**

|  | 0-50 | 50-100 | 100-150 | 150-200 | 200-250 | 250-300 |
| --- | --- | --- | --- | --- | --- | --- |
| Number of spines | 310 | 1107 | 2727 | 2407 | 508 | 148 |

The spines were ascribed to sections of 50µm long, from 0µm (beginning of the dendrite) to 300µm.
