# Supplementary material for: 3D morphology-based clustering and simulation of human pyramidal cell dendritic spines
Source: PLoS Comput Biol. 2018 Jun 13;14(6):e1006221. doi: 10.1371/journal.pcbi.1006221 (PMC6060563; doi:10.1371/journal.pcbi.1006221)
Supplement: S1 Text — Plain discriminative rules and summary statistics of the features. (DOCX) [file pcbi.1006221.s004.docx]

# Supporting Information

## S1 Text

#### Cluster 1: 92.19% spines correctly classified

($|h_{2}|$ ≤ 0.09667589)

#### Cluster 2: 84.49%

($V_{7}$ ≤ 0.01888145) and ($cos(\phi_{4})$ ≤ -0.9492174) and ($cos(\phi_{5})$ ≤ -0.964604)

#### Cluster 3: 75.62%

($\varphi_{46}$ ≥ 0.6491841) and ($\varphi_{46}$ ≤ 2.028975) and ($cos(\phi_{2})$ ≤ -0.9235236) and ($V$≤ 0.5182492)

#### Cluster 4: 84.48%

($V_{4}$ ≥ 0.1093572) and ($\varphi_{46}$ ≤ 0.8391926)

#### Cluster 5: 84.18%

($\varphi_{46}$ ≥ 0.8920209) and ($|h_{2}|$ ≥ 0.3030611)

#### Cluster 6: 89%

($V_{7}$ ≥ 0.09488738)

A set of tables summarizing the 36 morphological features that represent morphological aspects of the dendritic spines are shown. It can be used to compare and analyze the rules of the section “Cluster interpretation and visualization”. Each table represents a concrete feature along all the spine regions. Note that these are the values before standardization.

|  | $\vert\boldsymbol{h}_{1}\vert$ | $\vert\boldsymbol{h}_{2}\vert$ | $\vert\boldsymbol{h}_{3}\vert$ | $\vert\boldsymbol{h}_{4}\vert$ | $\vert\boldsymbol{h}_{5}\vert$ | $\vert\boldsymbol{h}_{6}\vert$ | $\vert\boldsymbol{h}_{7}\vert$ |
| --- | --- | --- | --- | --- | --- | --- | --- |
| Min | 9e-07 | 2e-05 | 5e-04 | 8e-03 | 0.02 | 0.03 | 0.02 |
| Q1 | 0.03 | 0.13 | 0.17 | 0.19 | 0.20 | 0.19 | 0.13 |
| Median | 0.10 | 0.23 | 0.26 | 0.26 | 0.26 | 0.26 | 0.18 |
| Mean | 0.12 | 0.24 | 0.26 | 0.27 | 0.27 | 0.27 | 0.20 |
| Q3 | 0.18 | 0.33 | 0.34 | 0.33 | 0.34 | 0.34 | 0.25 |
| Max | 0.95 | 1.21 | 1.13 | 1.16 | 1.15 | 1.13 | 1 |

|  | $B_{2}^{R}$ | $B_{3}^{R}$ | $B_{4}^{R}$ | $B_{5}^{R}$ | $B_{6}^{R}$ | $B_{7}^{R}$ |
| --- | --- | --- | --- | --- | --- | --- |
| Min | 0.03 | 0.04 | 0.04 | 0.06 | 0.05 | 0.03 |
| Q1 | 0.15 | 0.23 | 0.29 | 0.33 | 0.34 | 0.27 |
| Median | 0.20 | 0.30 | 0.35 | 0.41 | 0.44 | 0.36 |
| Mean | 0.21 | 0.31 | 0.37 | 0.42 | 0.46 | 0.39 |
| Q3 | 0.26 | 0.37 | 0.44 | 0.51 | 0.55 | 0.48 |
| Max | 0.67 | 0.82 | 0.94 | 1.09 | 1.24 | 1.16 |

|  | $B_{2}^{r}$ | $B_{3}^{r}$ | $B_{4}^{r}$ | $B_{5}^{r}$ | $B_{6}^{r}$ | $B_{7}^{r}$ |
| --- | --- | --- | --- | --- | --- | --- |
| Min | 0.02 | 0.03 | 0.03 | 0.04 | 0.03 | 0.03 |
| Q1 | 0.11 | 0.15 | 0.17 | 0.2 | 0.21 | 0.16 |
| Median | 0.15 | 0.19 | 0.22 | 0.26 | 0.27 | 0.21 |
| Mean | 0.15 | 0.2 | 0.23 | 0.27 | 0.28 | 0.22 |
| Q3 | 0.19 | 0.24 | 0.28 | 0.32 | 0.34 | 0.27 |
| Max | 0.42 | 0.54 | 0.76 | 0.78 | 0.79 | 0.70 |

|  | $\varphi_{24}$ | $\varphi_{26}$ | $\varphi_{46}$ | $V$ |
| --- | --- | --- | --- | --- |
| Min | 0.04 | 0.02 | 0.01 | 2e-03 |
| Q1 | 1.32 | 0.83 | 0.45 | 0.18 |
| Median | 1.85 | 1.25 | 0.65 | 0.35 |
| Mean | 2.32 | 2.05 | 0.93 | 0.46 |
| Q3 | 2.62 | 2.27 | 1.02 | 0.62 |
| Max | 80.31 | 84.21 | 37.22 | 3.98 |

|  | $cos(\phi_{1})$ | $cos(\phi_{2})$ | $cos(\phi_{3})$ | $cos(\phi_{4})$ | $cos(\phi_{5})$ | $cos(\phi_{6})$ |
| --- | --- | --- | --- | --- | --- | --- |
| Min | -1.00 | -1.00 | -1.00 | -1.00 | -1.00 | -1.00 |
| Q1 | -0.98 | -0.99 | -0.99 | -0.99 | -0.99 | -0.98 |
| Median | -0.95 | -0.96 | -0.97 | -0.98 | -0.98 | -0.95 |
| Mean | -0.86 | -0.90 | -0.93 | -0.95 | -0.96 | -0.90 |
| Q3 | -0.85 | -0.89 | -0.91 | -0.94 | -0.96 | -0.88 |
| Max | 0.99 | 0.99 | 0.61 | 0.88 | 0.61 | 0.98 |

|  | $V_{1}$ | $V_{2}$ | $V_{3}$ | $V_{4}$ | $V_{5}$ | $V_{6}$ | $V_{7}$ |
| --- | --- | --- | --- | --- | --- | --- | --- |
| Min | 0 | 5e-06 | 3e-04 | 7e-04 | 5e-04 | 2e-04 | 3e-05 |
| Q1 | 2e-03 | 0.02 | 0.03 | 0.05 | 0.06 | 0.04 | 0.01 |
| Median | 6e-03 | 0.04 | 0.06 | 0.09 | 0.11 | 0.09 | 0.02 |
| Mean | 9e-03 | 0.05 | 0.08 | 0.11 | 0.15 | 0.16 | 0.03 |
| Q3 | 0.01 | 0.07 | 0.11 | 0.15 | 0.20 | 0.18 | 0.04 |
| Max | 0.16 | 0.78 | 0.94 | 1.37 | 1.70 | 1.48 | 0.37 |
